# Supplementary material for: Driver Genetic Mutations in Spinal Cord Gliomas Direct the Degree of Functional Impairment in Tumor-Associated Spinal Cord Injury
Source: Cells. 2021 Sep 24;10(10):2525. doi: 10.3390/cells10102525 (PMC8533877; doi:10.3390/cells10102525)
Supplement: Supplementary file 1 [file cells-10-02525-s001.zip › cells-1339300-supplementary.pdf]

Supporting information

Driver genetic mutations in spinal cord gliomas direct the degree of functional impairment in tumor-associated spinal cord injury

Yoshitaka Nagashima[1], Yusuke Nishimura[1], Fumiharu Ohka[1], Kaoru Eguchi[2], Kosuke Aoki[1], Hiroshi Ito[1], Tomoya Nishii[1], Takahiro Oyama[1], Masahito Hara[3], Yotaro Kitano[1,4], Hirano Masaki[1,5], Toshihiko Wakabayashi[1], Atsushi Natsume[1+]

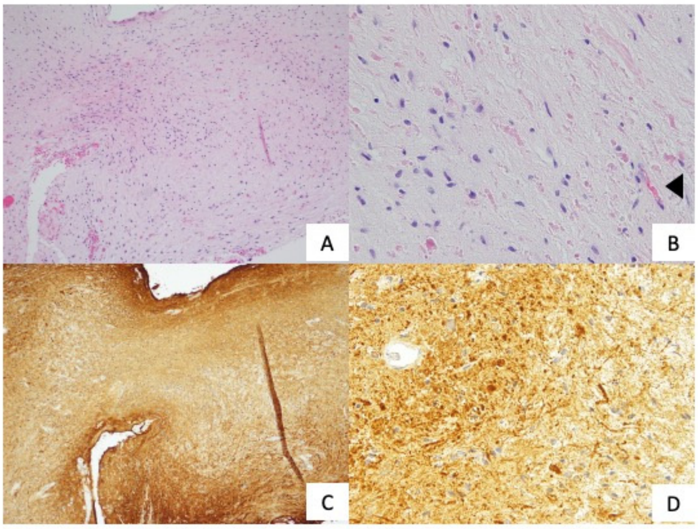

Supplementary Figure S1. Pathological images of WHO grade I pilocytic astrocytoma (Case #2): Pathological images showed alternating compact and loose growth patterns which called biphasic pattern (A: Hematoxylin and eosin stain, X100). Thick, elongated, eosinophilic, corkscrew-shaped rosenthal fiber was found in the compact regions (B: Hematoxylin and eosin stain, X400). GFAP (Glial fibrillary acidic protein) is positive in tumor cells (C: GFAP, X100, D: GFAP, X400).
